# A Novel Mouse Model of Type 2 Diabetes Using a Medium–Fat Diet, Fructose, and Streptozotocin to Study the Complications of Human Disease

**DOI:** 10.21203/rs.3.rs-5920886/v1

**Published:** 2025-03-12

**Authors:** Yanina Luciana Mazzocco, Gastón Bergero, Sebastián Del Rosso, Zoé M Cejas Gallardo, Alejandra Canalis, Ruth Eliana Baigorri, Luciana Mezzano, Juan Javier Mladin, Gustavo Tomas Diaz, Claudia Erika Martinez, Roxana Carolina Cano, Maria Pilar Aoki

**Affiliations:** Consejo Nacional de Investigaciones Científicas y Técnicas (CONICET), Centro de Investigaciones en Bioquímica Clínica e Inmunología (CIBICI); Consejo Nacional de Investigaciones Científicas y Técnicas (CONICET), Centro de Investigaciones en Bioquímica Clínica e Inmunología (CIBICI); Consejo Nacional de Investigaciones Científicas y Técnicas (CONICET), Centro de Investigaciones en Bioquímica Clínica e Inmunología (CIBICI); Consejo Nacional de Investigaciones Científicas y Técnicas (CONICET), Centro de Investigaciones en Bioquímica Clínica e Inmunología (CIBICI); Consejo Nacional de Investigaciones Científicas y Técnicas (CONICET), Centro de Investigaciones en Bioquímica Clínica e Inmunología (CIBICI); Consejo Nacional de Investigaciones Científicas y Técnicas (CONICET), Centro de Investigaciones en Bioquímica Clínica e Inmunología (CIBICI); Instituto de Biología Celular, Facultad de Ciencias Médicas, Universidad Nacional de Córdoba; Hospital Nuestra Señora de la Misericordia-Nuevo Siglo; Instituto de Biología Celular, Facultad de Ciencias Médicas, Universidad Nacional de Córdoba; Hospital Nuestra Señora de la Misericordia-Nuevo Siglo; Consejo Nacional de Investigaciones Científicas y Técnicas (CONICET), Centro de Investigaciones en Bioquímica Clínica e Inmunología (CIBICI); Consejo Nacional de Investigaciones Científicas y Técnicas (CONICET), Centro de Investigaciones en Bioquímica Clínica e Inmunología (CIBICI)

## Abstract

The study of type 2 diabetes mellitus (T2DM) pathophysiology relies mainly on the use of animal models, the most common of which involves the consumption of high-fat diets comprising 60% calories from fat. Although these models reproduce the onset and most complications associated with T2DM, they do not accurately mimic human dietary patterns, as they lack the addition of carbohydrates such as fructose. This study aimed to develop a C57BL/6 mouse model of T2DM that mimics the disease, as occurs in younger individuals, via a medium-fat diet (34.5% kcal from fat) combined with a 20% fructose solution as drinking water and a single low-dose of streptozotocin (STZ) (100 mg/kg), a diabetogenic drug. At week 20, D + T mice exhibited significant weight gain and elevated fasting blood glucose levels compared with those of control mice and the development of insulin resistance. Similarly, the circulating levels of hepatic enzymes (GPT, GOT, and alkaline phosphatase), total cholesterol, and LDL increased. Multi-organ damage, including reduced pancreatic islet size and number, severe hepatic steatosis, inflammatory infiltration in visceral adipose tissue, and cardiac and renal dysfunction, were also detected. The proposed model replicates T2DM in young mice by combining a medium-fat diet with fructose and STZ.

## Introduction

Diabetes mellitus (DM) is one of the most important global health threats of the 21st century and represents a major public health challenge, significantly impacting patient quality of life and survival. It is estimated that 537 million individuals worldwide lived with DM in 2022, and this number is projected to rise to 643 million by 2030^1^. Type 2 DM (T2DM) is a complex and heterogeneous metabolic disorder characterized by varying degrees of insulin resistance and insulin production deficiency, both of which contribute to the onset of hyperglycemia. This type of DM constitutes approximately 90% of cases globally. Notably, recent data show a rapid increase in T2DM during adolescence and early adulthood^2^. T2DM is closely associated with secondary complications arising from chronic hyperglycemia, including neuropathy, nephropathy, retinopathy, and increased risk of cardiovascular disease (CVD)^3^. While numerous studies have demonstrated that T2DM significantly increases susceptibility to infections^4–6^ and is a strong predictor of mortality related to infection^7^, the exact mechanisms underlying this enhanced vulnerability remain incompletely elucidated. Animal models in young individuals can provide invaluable insights into the pathophysiology of T2DM complications, the mechanisms driving susceptibility to infections, and the development of new diagnostic and therapeutic strategies for Young people with diabetes.

The study of T2DM pathophysiology relies mainly on the use of animal models. Presently, these experimental models consist of a broad variety of settings differing in the choice of animal species and the methodological approaches used to induce the disease^8,9^. A common approach involves the use of diets with 60% of calories derived from fats, which are effective in inducing T2DM features such as dyslipidemia, insulin resistance, inflammation, and nonalcoholic fatty liver disease (NAFLD), among other conditions^9,10^. This model attempts to mimic the Western diet (WD), which is characterized by excessive consumption of saturated fats. Nonetheless, this approach seems to fail to accurately mimic WD in humans, as it relies on excessive fat consumption while overlooking the intake of carbohydrates, such as fructose. Indeed, WD contains sugars such as high-fructose corn syrup (HFCS), which is commonly found in processed foods^11^. In this context, a recent prospective study reported a significant association between the consumption of sugary drinks and increased mortality rates and CVD incidence in T2DM patients^12^. These findings suggest that sugar intake may play a pivotal role in the development of T2DM and its associated comorbidities.

This study was designed to generate a novel model of T2DM in young mice that accurately replicates human disease and enables the use of genetic tools to deepen the understanding of its etiopathogenesis. This model combines a diet of medium-fat dry food with a 20% fructose solution as drinking water, along with a single low dose of streptozotocin (STZ), a diabetogenic drug. Detailed histological changes, functional abnormalities, plasma damage biomarkers, and circulatory lipid profiles were analyzed after the induction of the proposed model.

## Materials and methods

### Ethics statement

All animal experiments were carried out with the approval of the animal handling and experimental procedures of the Institutional Committee for the Care and Use of Laboratory Animals (CICUAL RD-2024-365-E-UNC-DEC#FCQ) of CIBICI-CONICET, Facultad de Ciencias Químicas, Universidad Nacional de Córdoba, Córdoba (Argentina), in strict accordance with the recommendation of the U.S. Department of Health and Human Services Guide for the Care and Use of Laboratory Animals. The study is reported in accordance with ARRIVE guidelines.

### Dietary conditions

#### 1. Experimental diet

Two commercial diets with different nutritional compositions were administered to the experimental groups: 1) the standard diet (29.4% protein, 56.2% carbohydrate, and 14.4% fat) was purchased from Asociación de Cooperativas Argentinas C.L., Buenos Aires, Argentina, and 2) the diabetes-inducing diet, a medium fat diet (MFD) (28.6% protein, 36.8% carbohydrate, and 34.5% fat), was obtained from TIT CAN GROSS S.A., Córdoba, Argentina. This group was additionally provided with drinking water supplemented with 20% w/v fructose (Biopack, Córdoba, Argentina). The nutritional composition is shown in Supplemental Information Table 1. The animals, which were grouped as described in the subsequent section, were allowed *ad libitum* access to their respective diets and water throughout the experimental procedure. Both water and food intake were monitored weekly, and daily caloric intake per mouse was calculated and adjusted proportionally (Table 1).

#### 2. Animals and experimental protocols

Six-week-old male C57BL/6J mice housed in the Animal Facility Unit of the Facultad de Ciencias Químicas, Universidad Nacional de Córdoba (registered with the NIH under OLAW-NIH F16-00193 (A5802-01)) were maintained under standard hygienic and environmental conditions, with a 12-hour light/dark cycle at 23°C ± 2°C and 50%–55% humidity. The mice were randomly divided into groups of three to five and fed either a standard diet, termed the “Control Diet,” identified as group “N,” or a “Diabetes-Inducing Diet,” identified as group “D,” for 20 weeks. The food was replaced every two days to ensure freshness. Body weight and general health status were monitored and recorded weekly. After 8 weeks of exposure to the diabetes-inducing diet, the animals were further divided into a “D+T” group receiving a single i.p. injection of 100 mg/kg STZ (Sigma CA) and a “D” group receiving vehicle (50 mM citrate buffer, pH 4.5)^13^. Seven days later, fasting glucose levels were measured by collecting a drop of blood from the tail. The measurements were performed via a OneTouch glucometer (Roche). D+T animals whose fasting glucose level was greater than 200 mg/dL were considered diabetic.

### Collection of Organs, Plasma, and Urine

At 20 weeks of treatment, to collect urine samples, the animals were placed in metabolic cages for 18 hours with free access to water and food. After urine collection, plasma samples were obtained through cardiac puncture under isoflurane anesthesia. The animals were perfused with cold phosphate-buffered saline (PBS) for organ collection. The organs were subsequently preserved for further processing.

### Histological determination

The pancreas, kidneys, heart, visceral adipose tissue (VAT), and a portion of the liver were fixed in 10% buffered formalin and embedded in paraffin. Five-micrometer-thick sections were examined under a light microscope (Nikon Eclipse TE 2000 U) after they were stained with hematoxylin and eosin (HE), PAS-hematoxylin, or Masson’s Trichrome. Ten random images from each tissue section were captured at 400x magnification for analysis. Quantification was performed via ImageJ software v. 1.41^14^. The Quantification of VAT histomorphometry was performed via ImageJ image analysis software with the “Adiposoft” plugin^15^.

### Tissue Fibrosis

Staining was performed as described by Hadi AM *et al*^16^. Briefly, a 0.1% Sirius Red F3BA solution in saturated aqueous picric acid was applied for 1 hour at 25°C. The sections were then washed in 0.01 N HCl for 2 minutes, dehydrated through a graded ethanol series, and cleared in xylene in two 10-minute stages. Finally, the sections were mounted with Canada balsam (Biopack). A minimum of 10 sections per tissue sample were analyzed at 20x magnification via polarization filters under a Nikon TE2000U microscope for Quantification. A threshold was applied to identify collagen-rich areas, followed by conversion to binary images^16^. The relative area occupied by collagen was calculated as a proportion of the total tissue area via ImageJ.

### Biochemical Determinations

The plasma samples were analyzed by the C.I.B.E.V Laboratory (Córdoba, Argentina) for glutamate–pyruvate transaminase (GPT), glutamate–oxaloacetate transaminase (GOT) and alkaline phosphatase (ALP) activity, as well as for total cholesterol, low–density lipoprotein (LDL) cholesterol, high–density lipoprotein (HDL) cholesterol, and triglyceride levels. Insulin levels were determined via a Mouse Ultrasensitive Insulin ELISA Kit (ALPCO) in accordance with the manufacturer’s protocol. The plasma determinations of urea and creatinine and the urine sample analyses for albuminuria, proteinuria, urea, and creatinine were conducted via an ARCHITECT c8000 analyzer (Abbott).

### Echocardiography and Electrocardiography

Echocardiographic (ECC) and electrocardiographic (ECG) measurements were obtained with the animals in a sedated state via a single dose (i.p.) of a mixture of xylazine (8 mg/kg) and ketamine (90 mg/kg), adjusted to maintain a heart rate between 200 and 400 bpm. Baseline electrocardiography (ECG) data were recorded via a noninvasive TM-300-V device (Temis Tech, Argentina) equipped with a four-electrode configuration and integrated with EasyG data acquisition software (Temis Tech, Argentina). Signal analysis was conducted via the software “*assisted measurement*” mode with the following settings: a sensitivity of 40 mm/mV, a recording speed of 50 mm/s, and filter adjustments set to 1 Hz for baseline correction, 75 Hz for bandwidth limitation, and 40 Hz for muscle noise reduction.

All echocardiograms were captured via LOGIQ ePRO R8 Color Doppler Echocardiography (General Electric) equipped with a 6.7 MHz. - 18.0 MHz mHz linear array transducer L8-18i-RS (General Electric). A nontoxic gel was applied to the probe, followed by recording of M-mode and B-mode echocardiograms. We used M-mode imaging in the parasternal long-axis and short-axis views to measure fractional shortening (FS). FS was calculated in both 2D mode and M mode by obtaining the maximum diastolic diameter and the minimum systolic diameter. The two-dimensional mode also allows us to calculate fractional shortening. We measured the end-diastolic and end-systolic diameters for both methods to perform the necessary calculations. The flow across the four cardiac valves was recorded via pulsed Doppler mode, and the velocity-time integral (VTI) for each heartbeat was calculated. In the apical five-chamber view, we observed the transmitral inflow and transaortic out flow. This enabled the determination of the systolic and diastolic isovolumetric times, as well as the total ejection time. These measurements were used to calculate the left ventricular myocardial performance index (LVMPI), also known as the Tei index.

### Statistical analysis

Data are presented as the means ± standard deviations for normally distributed variables or as medians with interquartile ranges (IQRs) for nonnormally distributed variables. The normality of distributions was assessed via the Shapiro–Wilk test and by inspecting Q–Q plots and box plots. Variables that did not exhibit a normal distribution after transformation were analyzed via nonparametric methods, including the Mann–Whitney U test and the Kruskal–Wallis test. All analyses were conducted with an alpha level of 0.05. Two-way ANOVA was used to analyze the changes in weight and glucose levels over time. Statistical analyses and figure creation were performed via Prism GraphPad v9.0 (GraphPad Software, San Diego, CA, USA).

## Results

### Effects on body weight, plasma glucose, insulin levels, and HOMA-IR

**Figure 1:**
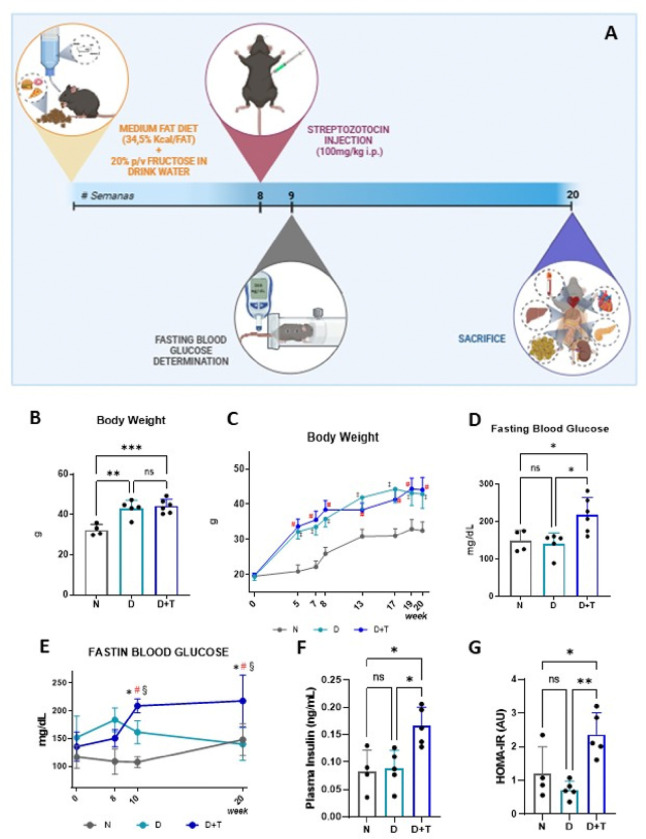
Description of the mouse model established after 20 weeks. **(A)** Experimental design. **(B-C-D)** Body weight and fasting blood glucose levels were determined at the end of the final week and at different time points. **(E)** Throughout the model time window. **(F)** Plasma insulin levels were determined by ELISA at week 20. **(G)** Homeostatic model assessment to evaluate insulin resistance at week 20. N (n= 4), D (n=5), D+T (n=5). B and D: * p ≤ .05; ** p < .01; *** p < .001; ns: nonsignificant. (C) #: D+T vs N at the same time point. †: N vs N t=0, ‡D vs N at the same time point. (E) * with respect to the point measured before in the same group, #: D+T vs N at the same time, §: D+T vs D+T t_*0*_.

The experimental protocol is depicted in Figure 1A. As shown in Figure 1B, the D and D+T groups presented significantly greater body weights than did the standard diet group (N) (D: 42.97 ± 4.08 g, D+T: 44.17 ± 3.45 g, N: 32.58 ± 2.43 g; D *vs*. N p= 0.0009, D+T *vs*. N p= 0.0002). These differences became evident from the fifth week and persisted until the endpoint of the study (Figure 1C) (time × group interaction, F_(14, 54)_ = 7.493, p<0.0001). Compared with the control animals or those receiving only the medium fat diet and fructose, the D+T group presented significantly greater blood glucose levels (D+T: 217.8 ± 46.8 mg/dL D: 140.2 ± 29.5 mg/dL; N: 148.3 ± 28.7 mg/dL; D+T *vs*. D p=0.0153; D+T *vs*. N p=0.0279). The differences were maintained throughout the study period (Figure 1E, time × group interaction, F_(6, 20)_ = 7.131, p=0,0004). Accordingly, at week 20, fasting plasma insulin levels were greater in the D+T group than in the N and D groups (Figure 1F), with no significant difference observed between these two groups. The homeostasis model assessment of insulin resistance (HOMA-IR) score was calculated via glucose and insulin data for all the groups. In the 20th week, the D+T group presented a higher HOMA-IR, indicative of greater insulin resistance, than the N and D groups did (Figure 1G). These results demonstrate that the proposed model effectively induces alterations in metabolic and hormonal homeostasis, resembling those observed in T2DM.

### Hepatic damage and lipid profiles

**Figure 2:**
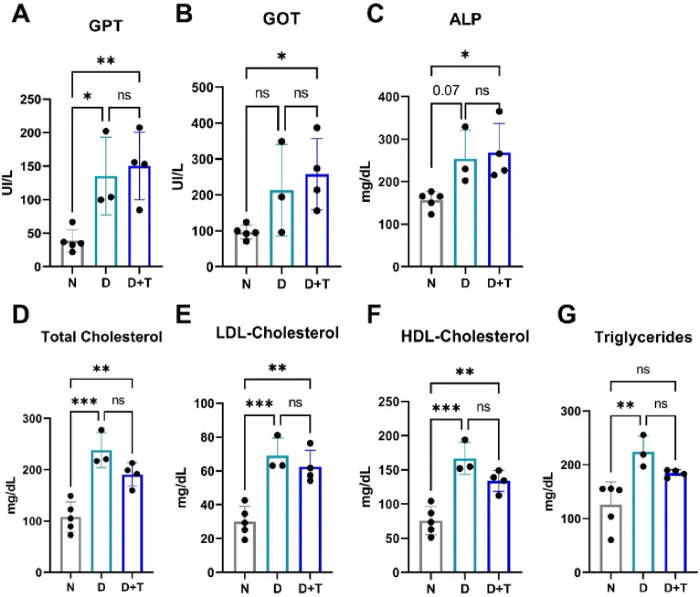
Effects of the diabetic model on plasma levels of hepatic enzymes and lipids. **(A)** Glutamate–pyruvate transaminase (GPT), **(B)** glutamate– oxaloacetate transaminase (GOT), **(C)** alkaline phosphatase (ALP), D–F) total, low-density lipoprotein (LDL) and high-density lipoprotein (HDL) cholesterol, and (E) triglycerides. N (n= 5), D (n=3), D+T (n=4). (*) p ≤ 0.05; (**) p < 0.01; ns: nonsignificant.

Compared with those in the N group, the plasma levels of the hepatic enzymes GPT, GOT and ALP in the D+T group were greater, indicating hepatic damage. Interestingly, the D group only presented an increase in GPT (Figure 2A–C). Additionally, total cholesterol, LDL, and HDL concentrations were significantly greater in the D and D+T groups than in the N group (Figure 2D–F). Notably, triglyceride levels were elevated in the D group compared with those in the N group, with no significant difference observed between the D and D+T groups (Figure 2G). These findings provide evidence that the proposed model can induce hepatic perturbations, such as NAFLD^17^ and dyslipidemia, which are frequently observed in individuals with T2DM and are strongly associated with increased susceptibility to CVD and immune disorders^18,19^.

### Histological analysis of selected tissues

**Figure 3:**
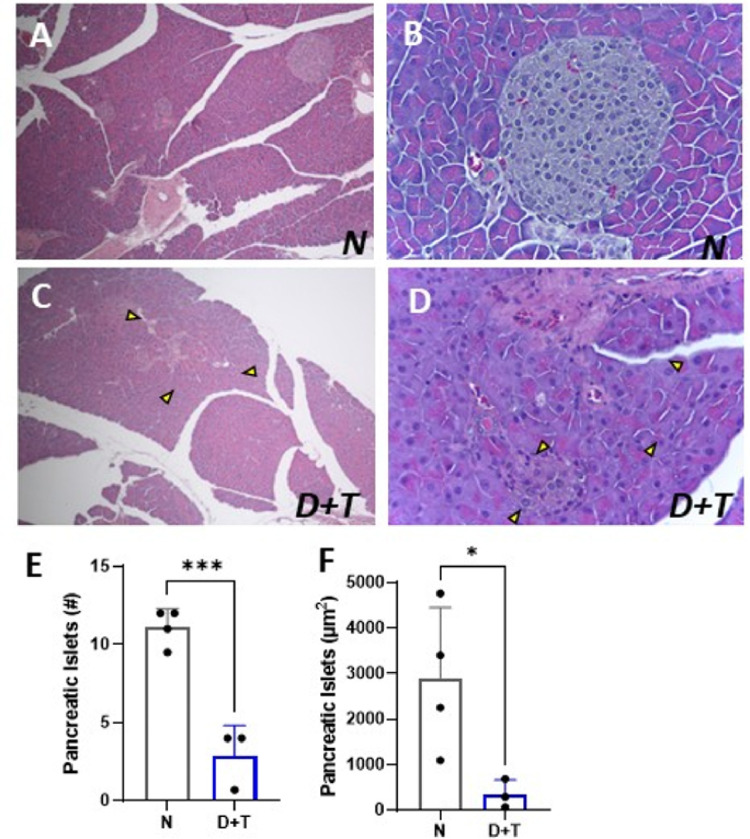
Histological analysis of the pancreas. **(A)** Nonmodel animal tissue (HE-10X), (B) islets of Langerhans with preserved morphology and size from animals in the N group (HE-40X), **(C-D)** pancreas of the D+T group. (*C*) Yellow arrow shows islets with altered morphology, loss of spherical shape, and reduced size (hypotrophy) (HE-10X). (*D*) Yellow marks indicate fibrosis, congestion and in filtration. (HE-40X). (**E**) Number of pancreatic islets evaluated per field, averaged over 10 fields per individual. (**F**) Average area of the evaluated islets. N (n= 4), D+T (n=3). (*) p ≤ 0.05; (***) p < 0.001.

To obtain a comprehensive understanding of the changes induced by the model, we performed a histological characterization of the pancreas, liver, and visceral adipose tissue (VAT). In contrast with the conserved structure in the pancreas of N group mice (Figure 3A–B), the pancreas of D+T group mice presented fat deposits and steatosis (data not shown), degenerative alterations, including large pyknotic acinar nuclei surrounded by a halo, reduced cellularity, and vascular congestion (Figure 3D), decreased islet size with altered morphology characterized by loss of spherical shape, reduced size, and number (Figure 3E–F). Moreover, this group exhibited signs of chronic or subacute pancreatitis, such as fibrosis, necrosis, and apoptosis. These results are consistent with the expected damage from the provided diet in combination with a single dose of STZ^20,21^.

**Figure 4:**
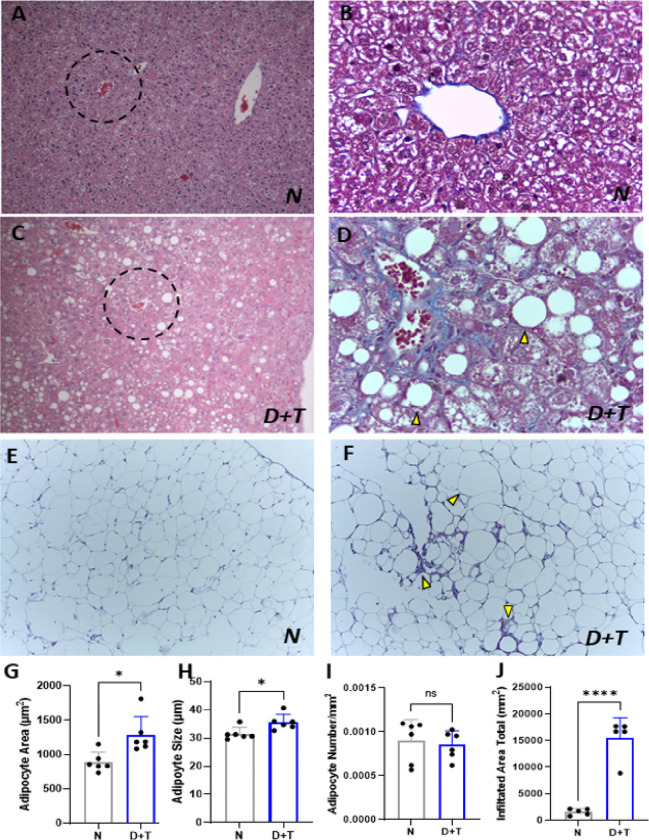
Histological analysis of liver and VAT. (**A**) Normal hepatic tissue (HE-10X). (**B**) Control mice exhibit relatively preserved hepatic morphology with mild steatosis. (**C-D**) Liver of the D+T group (Masson’s trichrome staining, 40X). (*C*) The livers of diabetic animals exhibited severe macro- and microvesicular steatosis, hepatocyte ballooning, congestion, and inflammatory infiltrates (HE-10X). (*D*) Yellow marks indicate fibrosis (pericellular and subsinusoidal) and steatosis. Congestion in the central vein and sinusoids, as well as a loss of normal hepatic architecture, can also be observed (HE-40X). (**E**) Nondiabetic VAT (HE-10X). (**F**) Diabetic VAT. The arrows indicate crown-like structures. (H-E 10X). (**G-H**) Adipocyte area and size in diabetic and control mice. (**I**) Number of adipocytes evaluated per mouse. (**J**) Area occupied by infiltrate per field, calculated via 10X magnification H-E staining. N (n= 6), D+T (n=6). (**) p<0.01; ns: not significant.

The livers of the N group mice presented a normal morphology characterized by the appropriate radial arrangement of hepatocytes around the central vein and well-organized sinusoidal spaces (Figure 4A–B). In contrast, the livers from the D+T group exhibited severe steatosis accompanied by uniform hepatocyte degeneration with ballooning and distorted arrangement of hepatic plates (hepatocellular injury), pericellular and subsinusoidal fibrosis, apoptosis in hepatic cells, and congested sinusoidal spaces (Figure 4C–D).

The VAT from D+T mice compared with that from control mice (Figure 4E) showed adipocyte hypertrophy without differences in the total number of adipocytes (hyperplasia) (Figure 4F–I) or crown-like structures (CLS) (Figure 4F–J), with an increase in the number of nonadipose cells in D+T VAT compared with that in the N group (D+T: 3.25×10^6^ ± 65.5×10^3^ cells; N: 1.7×10^6^± 81.8×10^3^ cells; p<0.0001).

**Figure 5:**
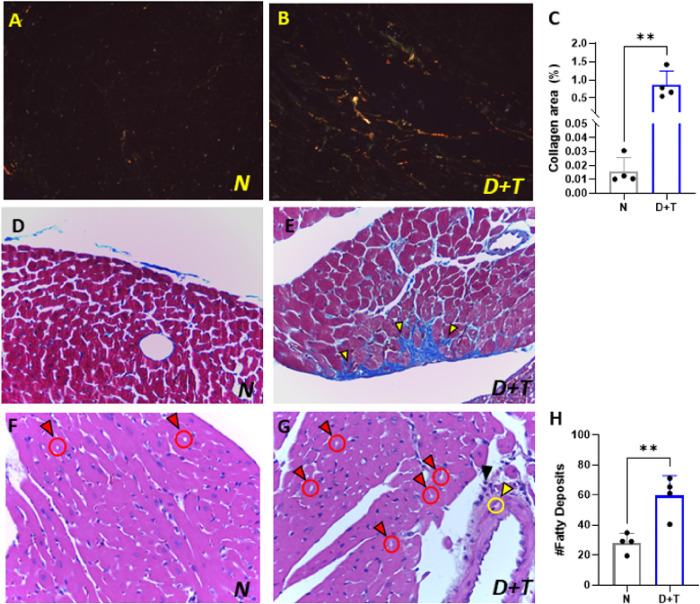
Histological inspection of cardiac tissue. Assessment of collagen accumulation in normal mice **(A)** and diabetic mice **(B)** via Picrosirius Red staining (upper panels) and under polarized light (lower panels), where reddish–pink hues are indicative of type I collagen fibers and green hues are indicative of type III collagen fibers. **(C)**Quantification of the percentage of collagen area in polarized images. **(D-E)** Histological evaluation of tissue via Masson’s trichrome staining. *(D)*Tissue sample from control group mice (N) (10x). *(E)* Cardiac tissue from D+T mice. Collagen deposits are indicated by yellow arrows. **(F-G, H)** Hematoxylin-stained cardiac tissue. *(F)* Cardiac explants from N mice. *(G)* Cardiac explants from the D+T group. Red arrows highlight lipid deposits within the myocardium, whereas yellow arrows indicate deposits in the muscular layer of the coronary artery. The black arrow indicates the presence of infiltration. *(H)*Fatty deposit Quantification. *N (n= 4), D+T (n=4)*. (*) p≤0,05; (**) p<0.01.

In cardiac tissue, the model induced an increase in fibrosis, as measured by Picrosirius Red and Masson’s Trichrome staining techniques (Figure 5A–E). Additionally, lipid deposits were observed in the myocardium of the animals in the D+T group and in the muscular tunica of the arteries (Figure 5F–H). Small infiltrates were also observed within the myocardium of this group. These results indicate that animals subjected to experimental models exhibit a low degree of myocarditis and active tissue repair processes.

### Assessment of cardiac function by echocardiography and electrocardiography

**Figure 6:**
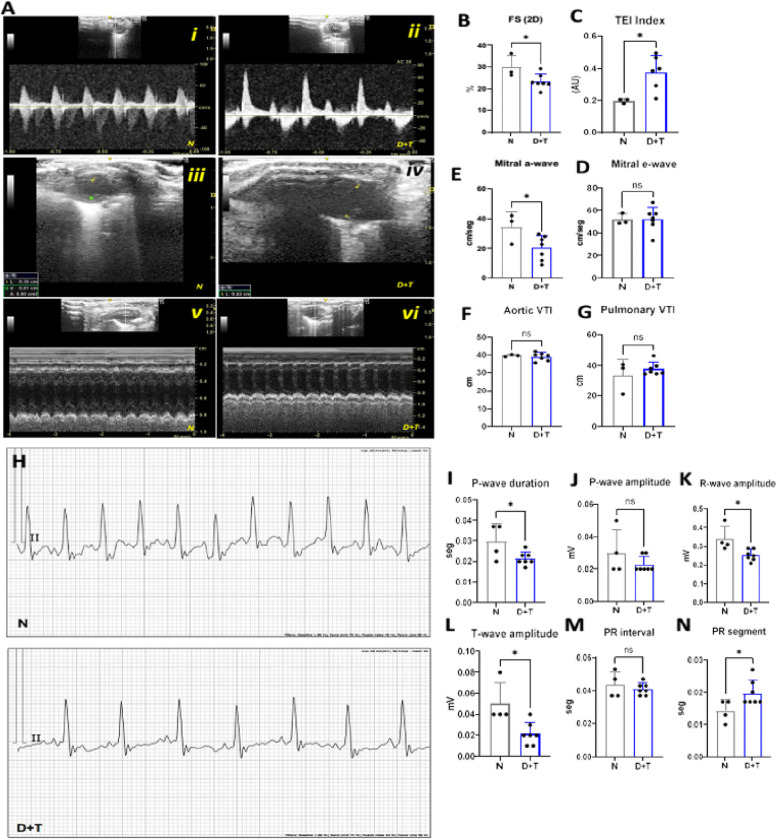
Cardiac function assessment. **(A-G)** Echocardiographic findings. *(A i-ii)* Apical 4-chamber view, inflow and outflow of the left ventricle. PVI Pulsed-wave Doppler. *(A iii-iv)* Parasternal long axis (PLAX) view - left ventricular diastolic dimension. *(A v-vi)* PLAX M-mode. *(B)* Fractional shortening, calculated as ((Diastolic Diameter - Systolic Diameter)/Diastolic Diameter)×100. *(C)* The TEI index (also known as the myocardial performance index, MPI) was calculated as (total ejection time - ejection time)/Ejection time. *(D-E)* Mitral E- and A-waves were obtained via Doppler echocardiographic waveforms. *(F-G)* Aortic and pulmonary velocity time integral (VTI) **(H-N)** ECG measurements. *(H)* Representative ECGs from the N (top) and D+T (bottom) groups of mice. *(I-J)* P-wave duration and amplitude. *(K–L)* R- and T-wave amplitudes. *(M-N)* PR interval and segment. *N (n= 3), D+T (n=7)*. (*) p≤0,05

Compared with N animals, D+T mice displayed signs of impaired cardiac functionality, as shown by lower 2D fractional shortening (FS) (Figure 6B). This feature is indicative of diminished systolic function^22,23^ and is often reported in cardiac fibrosis models^24^ and ischemia. These results align with the fibrosis deposits described. (Figure 5B, E). In line with this, D+T animals also exhibited decreased R- and T-wave amplitudes on ECG (Figure 6K–L), suggesting a reduction in action potential associated with a lower number of depolarized cells. These findings may contribute to diminished ventricular contractility, given that these waves reflect the electrical activity of ventricular depolarization (R) and repolarization (T)^25^. Notably, one of the most significant findings was the increased TEI index (LVMPI) observed in D+T animals (Figure 6C), a critical marker of both systolic and diastolic dysfunction^26^, indicating overall cardiac impairment. The increase in the TEI index, along with other indicators, such as reduced 2D FS, suggests an overall decline in ventricular function in this diabetic model. Additionally, an increase in the PR segment (Figure 6N) suggests possible atrioventricular conduction delay or blockage^27^. This change has been linked to an increased risk of mortality in patients with ST-elevation myocardial infarction (STEMI)^28^. Compared with the N group, the D+T group presented a reduction in the number of mitral A waves (Figure 6E).

The mitral A wave represents the atrial contraction phase during ventricular filling^29^. Therefore, the decrease in this parameter suggests impaired ventricular filling during atrial contraction. The reduction in the mitral A-wave, combined with an increase in the TEI index, suggests diastolic dysfunction with a pseudonormal pattern. However, this conclusion is limited by technical constraints, such as the inability to assess tricuspid valve regurgitation, tissue Doppler imaging, or tricuspid insufficiency. Despite these limitations, the observed alterations align with diastolic dysfunction typically linked to myocardial damage, including ischemia or fibrosis. Finally, in D+T mice, a shorter P-wave duration with no difference in P-wave amplitude was observed (Figure 6I). This may indicate early ventricular alterations and an elevated risk for atrial fibrillation^30^. No change was observed in aortic or pulmonary VTI (Figure 6F–G) or in the QT interval (data not shown). Together, these findings reveal both electrical conduction and mechanical function abnormalities in the heart, suggesting diabetic cardiomyopathy with concurrent systolic and diastolic dysfunction that aligns with the physiopathology described in the histopathology fibrosis analysis.

### Evaluation of Renal Alterations

**Figure 7:**
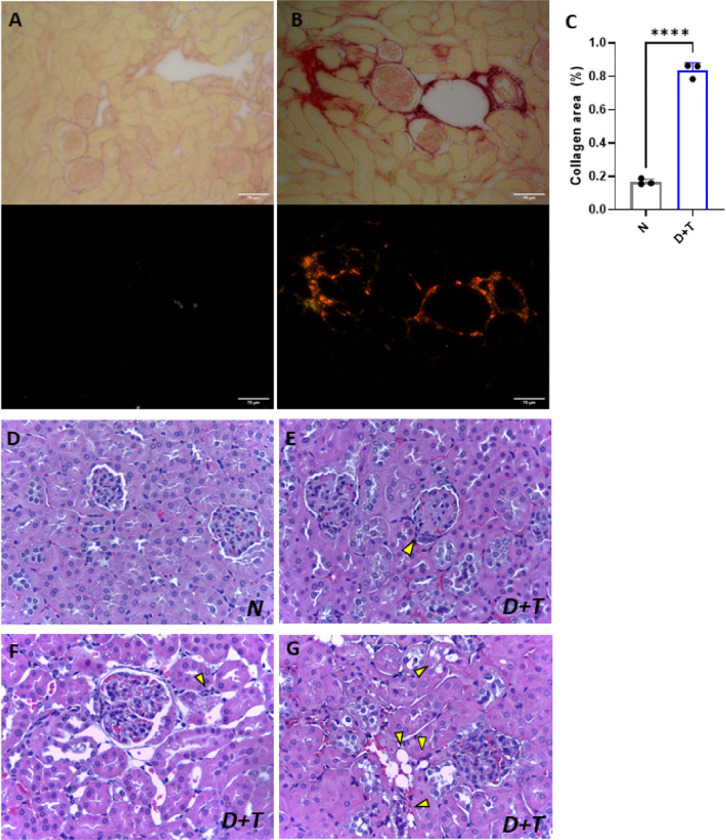
Renal histological analysis. **(A-C)** Assessment of collagen accumulation in normal mice *(A*) and diabetic mice *(B)* via Picrosirius Red staining (upper panels) and under polarized light (lower panels), where reddish–pink hues are indicative of type I collagen fibers and green hues are indicative of type III collagen bers. *(C)*Quantification of the collagen area in polarized images. (**D-G)**Histological evaluation of tissue via H&E staining. *(D)* Proportion of healthy tissue from control animals (10x). (**E-G**) Slices of renal tissue from D+T mice. Yellow arrows indicate segmental sclerosis in the glomeruli (*E)*, cellular infiltrate *(F),* steatosis and vascular congestion *(G)* (40X). N (n= 3), D+T (n=3). (****) p <0.0001.

Signs of renal impairment in D+T mice were characterized by a significant decrease in urine excretion over an 18-hour period, coupled with a decline in the glomerular filtration rate exceeding 50%, even in the D group (**Supplementary Figure 1A-B**). Moreover, the D+T group presented increased albuminuria (**Supplementary Figure 1C**), without changes in urinary creatinine or protein (**Supplementary Figure 1D-E).** Therefore, the D and D+T animals presented a reduction in urinary urea levels (**Supplementary Figure 1F**). Remarkably, D+T mice presented a higher plasma creatinine ratio than did N mice, which is indicative of microalbuminuria (**Supplementary Figure 1G**). Neither the D nor the D+T group presented differences in plasma urea levels compared with those of the N group (**Supplementary Figure 1H**). In line with these findings, the D+T group presented increased plasma creatinine levels compared with those of the control group, whereas no changes were observed in the D group (**Supplementary Figure 1I**). Histologically, there was more fibrosis in the glomerular and interstitial regions in the D+T group (**Supplementary Figure 7A-C**), which included glomeruli with segmental sclerosis and diffuse mesangial expansion accompanied by hypercellularity (Figure 7D–E), focal inflammatory infiltrates (Figure 7F), tubular vacuolization (hydropic degeneration), steatosis, and vascular congestion (Figure 7G). These data revealed a significant loss of renal functionality in the experimental model.

## Discussion

The present study aimed to develop and characterize a mouse model that mimics the natural progression and metabolic features of human type 2 diabetes by employing a medium-fat diet and fructose in the drinking water. Our long-term goal is to develop a model for in-depth immunological studies and pharmacological screening in young mice. Our results demonstrated that a combination of a medium-fat diet and fructose with a single dose of STZ allowed the development of experimental T2DM associated with fasting hyperglycemia, insulin resistance, dyslipidemia, and substantial signs of tissue damage. Moreover, the schema was found to induce increased weight gain and T2DM-associated complications such as hepatic steatosis and CVD, as evidenced by cardiac dysfunction, lipid deposition in the myocardium and chronic kidney failure.

Currently, the impact of fructose consumption on the development of T2DM is well established. Compelling evidence from various clinical trials and observational studies suggests that the addition of fructose, in the form of HFCS, increases the risk of T2DM^31–33^. Augmented fructose intake may be one of the major changes in food consumption in modern society. Between the 1970s and 2000s, the average annual consumption of HFC in the US increased dramatically from 0.23 kg to 28.4 kg^34^. In this context, an 8-year prospective cohort study reported that individuals who consumed more than one sweetened beverage per day had an 83% greater risk of developing T2DM than those who drank fewer than one beverage per month^35^. Therefore, fructose is an essential and irreplaceable factor to consider when developing an in vivo model of T2DM that accurately reflects the pathophysiology of the disease in humans^9^. In our model, fructose was administered in drinking water to mimic fructose intake in beverages.

Among the pathologies related to chronic fructose intake, NAFLD represents the most prevalent disease. NAFLD is defined by the presence of hepatic steatosis and lobular inflammation with no evidence of infection, inborn metabolic disorder, intake of steatogenic drugs, or chronic alcohol consumption^38^. In our model, mice subjected to the diabetogenic diet exhibited histological changes compatible with NAFLD, with incipient inflammatory focus and tissue damage. Strikingly, lipid deposition was also detected in the pancreas, kidney, and media of smooth muscle cells in artery walls. This finding is consistent with increasing evidence indicating that fructose consumption, especially in liquid form, is linked to the development of NAFLD^35^.

Another relevant aspect of the proposed model is the fact that T2DM and concomitant NAFLD development are achieved by employing a medium-fat diet (34.5% kcal of fat), in contrast with the most common approach, which uses a high-fat diet (60% kcal of fat)^9^. Reducing the amount of fat administered is particularly important for gathering reliable data in immunological studies. The rate and nature of fat circulation, as well as the composition of adipose tissue, directly influence immunometabolism and, consequently, the immune response to infections^36^. This immunology area is rapidly expanding and has the potential to lead to highly promising therapeutic intervention strategies. Consequently, lower fat intake in animal models of T2DM should be considered an important strategy for obtaining more reliable data during immunological studies.

In our study, a medium-fat diet and fructose-enriched water were combined with a single dose of STZ to reduce the pancreatic β-cell mass, thereby decreasing insulin production and mimicking the pathophysiology of human T2DM. This strategy reduces the time required to induce diabetes, thereby overcoming one of the major limitations of this approach. In this regard, our model resulted in a significantly lower number of islets in D+T mice with concomitant pancreatic amyloid deposition. As such, the results obtained recapitulate the major pathophysiological aspects of T2DM in this target tissue. Furthermore, reducing the time required to develop diabetic clinical features will enable immunological studies to be conducted during adolescence and early adulthood^36^, as age significantly alters individual inflammatory states and immune responses. In this sense, it is important to stress that the prevalence of T2DM in adolescents and young adults is increasing rapidly. Among the causes of this phenomenon are the sedentary lifestyle adopted in recent decades and the increased prevalence of obesity in childhood and adolescence, which parallels trends in the incidence of T2DM^37^. The increasing prevalence of T2DM in adults could also be linked to a greater risk of diabetes in descendants due to in-utero exposure to diabetes^38^. Accumulating evidence shows that the young onset of T2DM is associated with a more aggressive disease phenotype, leading to the premature development of complications with unfavorable effects on long-term outcomes^39^. This has challenged the scientific community in the development of experimental models for studying this disease during this stage of life.

Finally, we selected mice to induce the T2DM model since mouse models have enabled breakthroughs in understanding the pathophysiology of many immune-mediated diseases. Indeed, genome sequencing, sophisticated strategies for gene manipulation to obtain conditional animals, and the ability to transfer cells from one inbred mouse to another without eliciting immunological rejection, among other strategies, have helped accelerate the application of mice to the investigation of human diseases in the immunology and pharmacological fields. Implementing the present model via experimental tools developed in mice will enable a deeper understanding of the disease process and facilitate the testing of potential therapeutic interventions.

Animal models have been essential players in advancing diabetes research. However, no animal model is capable of fully recapitulating the human type 2 diabetic phenotype. This underscores the critical need for ongoing research efforts to improve these experimental models. The combination of fructose and a medium–fat diet with a single dose of STZ in C57BL/6 mice is associated with all the major pathophysiological aspects of T2DM. The model would be useful as a tool for studying immunological aspects in the setting of this disease and for developing more effective and tailored treatments, ultimately aiming to improve the quality of life for individuals living with diabetes.

## Figures and Tables

**Table 1:** Calculation of Daily Caloric Intake per Mouse (%Kcal)

**Proteins**	29,4%	21,4%
**Available Carbohydrates** *	56,2%	52,8%
*Fructose* **	-	*25,3%*
**Fat**	14,4%	25,8%

* Provided by food + fructose-supplemented water.

** Provided by the fructose-supplemented water.

## Data Availability

The datasets generated during and/or analyzed during the current study are available from the corresponding author upon reasonable request.
